# Multi-Modal Temporal Hypergraph Neural Network for Flotation Condition Recognition

**DOI:** 10.3390/e26030239

**Published:** 2024-03-08

**Authors:** Zunguan Fan, Yifan Feng, Kang Wang, Xiaoli Li

**Affiliations:** 1 Faculty of Information Technology, Beijing University of Technology, Beijing 100124, China; fzg100518@163.com (Z.F.); lixiaolibjut@bjut.edu.cn (X.L.); 2 School of Software, Tsinghua University, Beijing 100084, China; evanfeng97@gmail.com

**Keywords:** flotation condition identification, MVResNet, temporal HGNN, multi-modal fusion, froth image sequence

## Abstract

Efficient flotation beneficiation heavily relies on accurate flotation condition recognition based on monitored froth video. However, the recognition accuracy is hindered by limitations of extracting temporal features from froth videos and establishing correlations between complex multi-modal high-order data. To address the difficulties of inadequate temporal feature extraction, inaccurate online condition detection, and inefficient flotation process operation, this paper proposes a novel flotation condition recognition method named the multi-modal temporal hypergraph neural network (MTHGNN) to extract and fuse multi-modal temporal features. To extract abundant dynamic texture features from froth images, the MTHGNN employs an enhanced version of the local binary pattern algorithm from three orthogonal planes (LBP-TOP) and incorporates additional features from the three-dimensional space as supplements. Furthermore, a novel multi-view temporal feature aggregation network (MVResNet) is introduced to extract temporal aggregation features from the froth image sequence. By constructing a temporal multi-modal hypergraph neural network, we encode complex high-order temporal features, establish robust associations between data structures, and flexibly model the features of froth image sequence, thus enabling accurate flotation condition identification through the fusion of multi-modal temporal features. The experimental results validate the effectiveness of the proposed method for flotation condition recognition, providing a foundation for optimizing flotation operations.

## 1. Introduction

Flotation, as an essential technology in the mineral processing industry, facilitates the extraction of metal minerals from ores by leveraging the contrasting hydrophilicity and hydrophobicity of mineral particles. The operational state of the flotation process has a direct influence on its efficiency. Therefore, the rapid and accurate identification of process conditions plays a crucial role in optimizing and controlling flotation operations. Nevertheless, the flotation production process is intricate, subject to numerous physical and chemical factors, constituting a complex industrial engineering system with characteristics such as nonlinearity, hysteresis, and strong coupling [1]. Consequently, systematically describing the froth characteristics within the flotation cell and achieving precise condition identification pose significant challenges. Presently, most flotation plants heavily depend on the expertise of operators, who rely on visual observations of the froth state to discern flotation conditions. However, this approach suffers from subjectivity, lacks standardized evaluation criteria, and has other inherent issues, preventing flotation operations from reaching their optimal state. Consequently, investigating intelligent methods grounded in machine learning and computer vision from froth video holds substantial importance in enhancing the efficiency of flotation condition recognition.

During the practical implementation of flotation in production processes, the visual attributes of slurry froth within the flotation cell, such as color, texture, and morphology, serve as direct indicators of the current working state. Extracting a comprehensive set of informative image features from froth is crucial for accurately classifying flotation conditions. Over the years, researchers have dedicated considerable effort to investigating and exploring various methods for extracting froth image descriptors. Improved watershed algorithms [2,3] are proposed to enhance segmentation precision and facilitate bubble size feature extraction. Regarding color feature extraction, Hatonen et al. [4] undertook an analysis of statistical data pertaining to color components within the RGB spectrum. To overcome the issues of computational intensity and low accuracy inherent in texture feature extraction, Chen et al. [5] devised the Color Co-occurrence Heterogeneity Structure (CCHS) method leveraging color space transformation and matrix manipulation, and Tang et al. [6] proposed a methodology based on Local Binary Patterns Variance (LBPV), which integrates local spatial structures with contrast measures to facilitate the extraction of texture features.

In addition to the static extraction methods mentioned above, the inherently dynamic nature of the flotation process, marked by continuous fluctuations, poses significant challenges for the accurate and swift extraction of dynamic froth characteristics. To tackle this issue, dynamic feature matching [7] and cumulative distribution statistics [8] strategies are introduced to extract dynamic features and encapsulate temporal attributes. Advancing the exploitation of temporal data on froth surfaces, Ai et al. [9] formulated a deep learning-based dual-flow feature extraction model dedicated to assessing froth appearance and movement characteristics. Focusing the volatility of dynamic texture, Zhang et al. [10] integrates local binary patterns with gray level co-occurrence matrix histograms, offering a more comprehensive encapsulation of both local and global texture details. Although these methods enhance the robustness of dynamic feature extraction, they are beset by increased computational demands and the challenges of managing extensive data volumes. Moreover, the task of elucidating certain feature correlations with the flotation process complicates their real-time implementation and the comprehensibility of model applications.

The operational state of the flotation process significantly influences the quality of target minerals. Traditional methods that subjectively categorize flotation conditions based on human experience are plagued by substantial inaccuracies and inefficiencies. The precise recognition of flotation conditions is vital for the effective optimization and management of the flotation process. Traditional methods based on human experience are gradually replaced by data-driven approaches to obtain accurate classification of flotation conditions. The weighted support vector machine [11] and its variant [12] are proposed to address the challenges posed by the diversity, significance variance, and uneven sample distribution of flotation froth visual characteristics, achieving effective recognition of flotation conditions.

To improve the classification of various operating conditions in flotation processes, the integration of deep learning and machine vision techniques has shown considerable promise. Zarie et al. [13] devised a convolutional neural network (CNN)-based methodology for the effective classification of flotation froth images, accommodating six and four classification scenarios to suit diverse operating conditions. Through the examination and comparison of pre-trained network architectures such as AlexNet, VGG16, and ResNet, Fu et al. [14] introduced strategies aimed at enhancing classification precision and expediting the training process. Leveraging the Inception ResNet V1 deep learning architecture coupled with the XGBoost algorithm, Xie et al. [15] combined depth vision and image features to attain precise froth image classification. Additionally, employing the autoregressive moving average (ARMA) model alongside the Mahalanobis distance criterion, Chen et al. [16] achieved accurate detection of flotation froth states across varying operational conditions, utilizing dynamic texture features of the froth.

Multi-modal machine learning facilitates the processing of pertinent information across various modalities, including images, text, audio, and video, for classification tasks. This approach also extends to a category of generalized multi-modal data, derived from extracting multiple feature sets from original datasets via diverse feature extraction techniques [17]. Liao et al. [18] utilizes dual-mode imagery (visible light and infrared) and multi-scale CNN features in conjunction with an adaptive depth self-coding kernel extreme learning machine (KELM) for the fusion of features and decision-making in high-dimensional spaces. Furthering this domain, Liang et al. [19] put forward a technique for characterizing flotation conditions that integrates froth highlight distribution with image grayscale and texture features and utilizes SVM for classification, thereby evidencing that the amalgamation of comprehensive features outperforms the reliance on singular features in classification tasks.

The flotation process is inherently complex and slow, characterized by the extrusion and adhesion of bubbles within the flotation cell [20], resulting in diverse variation trends in froth movement. Most of the features extracted by the above method are static statistical features or dynamic features extracted under single mode, which limits the comprehensiveness and richness of froth image description. Additionally, the fusion of characteristic data from multiple modes often relies on simple linear splicing, disregarding the correlation between data samples. Consequently, there is an urgent need to enhance the accuracy of flotation working state recognition. Recently, hypergraph neural networks (HGNNs) have been widely used in tasks such as image classification [21] and retrieval [22]; leveraging its established capability in addressing intricate high-order correlations among data [23], the HGNN was introduced to identify flotation conditions through the utilization of manually extracted texture features, thereby achieving a high level of performance [24].

**Figure 1:**
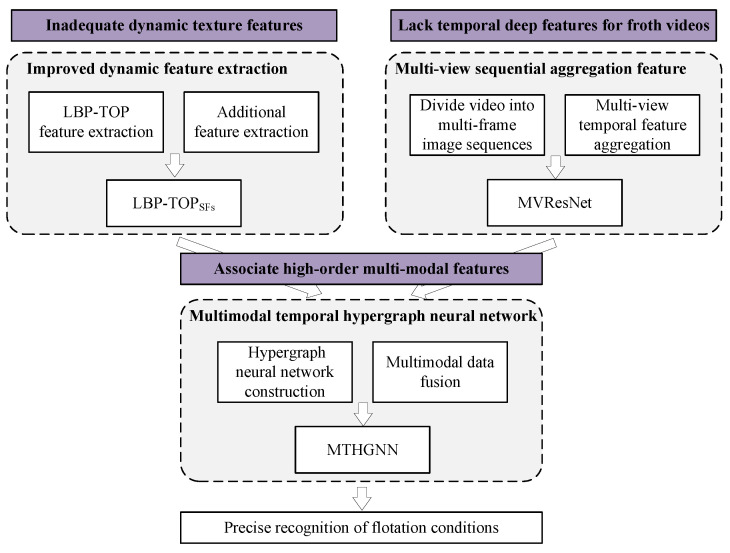
Research content sketch.

Inspired by the above-mentioned work, the motivation of this paper is to employ the HGNN and various methods to integrate time dynamic information into the feature extraction process and fuse extracted multi-modal temporal features to further enhance the accuracy in working condition classification. The main contributions are depicted in Figure 1 and summarized as follows:

- To enrich the dynamic texture features, a novel dynamic froth feature extraction algorithm, integrating local binary mode based on three orthogonal planes (LBP-TOP) and supplementary features, is introduced. The algorithm orthogonalizes the froth map sequence into three directions according to the spatio-temporal relationship, enabling the extraction of dynamic texture features containing spatial and temporal information along the three directions. In addition, froth description features such as kurtosis, skewness, and coefficient of variation are extracted, respectively, in each direction and then linearly fused with LBP-TOP to further enrich the froth dynamic description information;
- A new method for extracting multi-view sequential aggregation ResNet features from froth videos is proposed. To leverage the temporal information within the flotation froth video effectively, each video is divided into multi-frame image sequences, treated as multiple views of the froth video. Subsequently, a multi-view temporal feature fusion network (MVResNet) is constructed, utilizing multi-channel ResNet as the foundation for pre-training and temporal feature extraction;
- To optimize the dynamic texture feature and temporal feature of froth images, a multi-modal temporal hypergraph neural network (MTHGNN) is constructed. This network encodes higher-order data by constructing hypergraphs, enabling the association of more abundant feature information and facilitating the flexible modeling of complex features. Additionally, this method achieves the fusion of multi-modal feature data through the splicing hypergraph adjacency matrices, thereby enhancing the description of froth images and further improving the recognition accuracy of flotation conditions.

The remainder of this article is organized as follows. Section 2 discusses the extraction of LBP features using the TOP approach, along with the addition of supplementary features. Section 3 describes the development of the MVResNet model for extracting temporal features. Section 4 explores the integration of multi-modal data through the MTHGNN. Section 5 and Section 6 are dedicated to the comparative analysis of experimental results and the concluding remarks, respectively.

## 2. Dynamic Texture Feature Extraction Based on LBP-TOP and SFs

Froth formation within flotation cells represents a complex, dynamic process governed by multifarious factors. The conventional Local Binary Patterns (LBP) operator, renowned for its simplicity, efficiency, and robustness to variations in illumination, serves as a local texture descriptor [25]. Nonetheless, when applied to single-frame froth imagery, the traditional LBP operator is limited to delineating static bubble characteristics, thereby failing to capture the temporal dynamics of froth behavior. Addressing this deficiency, the LBP-Three Orthogonal Planes (LBP-TOP) algorithm is proposed, which innovatively extends the analysis of froth maps from a two-dimensional to a three-dimensional domain through the integration of LBP methodology and the TOP technique. This advancement allows the LBP-TOP algorithm to assimilate both spatial and temporal data, thus proficiently extracting temporal features from sequences of froth images, a capability that is critical for the dynamic analysis of froth behaviors in flotation cell environments. Building on this foundation, features of kurtosis, skewness, and coefficient of variation in three directions were extracted to supplement, thereby enriching the descriptive nature of the froth images.

### 2.1. Local Binary Patterns

Initially proposed by Ojala et al., LBP is a widely used gray-scale image descriptor for capturing local texture features [26]. LBP has gained prominence in applications like micro-expression analysis and face recognition due to its ability to discriminate texture features in images and its robustness to variations in illumination and posture. The LBP algorithm operates by considering a circular neighborhood with a specific radius within the image, which expands the range of the neighborhood and employs bi-linear interpolation to enhance pixel expansion, thereby enhancing the robustness of the LBP operator. A set of binary numbers is obtained by threshold operation between the gray values of the *P* pixels around the central pixel $\left(x_{c},y_{c}\right)$ in the field of *R* radius and the gray values of the central pixel, and then the LBP characteristic values of the central point are obtained. Equation (1) describes the calculation of the LBP characteristic value for the central pixel [27].

(1) $$\mathrm{LB}P_{P,R}\left(I\right)=\sum_{p=0}^{P-1}\mathrm{sign}\left(g_{p}-g_{a}\right)2^{p}.$$

where $g_{a}$ represents the gray value of the local central pixel in image *I*, $g_{p}$ represents the gray value of the local *p*th pixel, and sign(·) is a symbolic function as shown in Equation (2). If the gray value of the surrounding pixel $g_{p}$ is not less than the gray value of the central pixel $g_{a}$, the local binary code of the pixel is set to 1. Otherwise, the local binary code of the pixel is set to 0.

(2) $$\mathrm{sign}\left(g_{p}-g_{a}\right)=\left\{\begin{array}{cc} 1, & g_{p}\leq g_{a}\\ 0, & g_{p}>g_{a} \end{array}\right..$$

**Figure 2:**
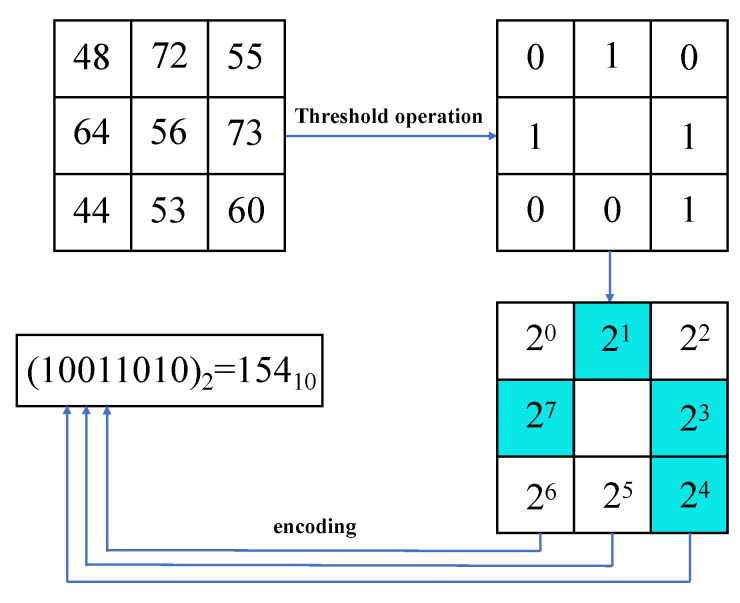
LBP schematic diagram.

The original LBP operator is defined in a rectangular neighborhood of 3×3. The gray-scale values of the central pixel and 8 adjacent pixels are compared, as shown in Figure 2. The surrounding 8-bit binary number is read clockwise and then converted into decimal, representing the LBP characteristic value of the current central pixel. The principle remains the same for the circular neighborhood, but in this case, the neighborhood radius and the number of equidistant pixels need to be set.

### 2.2. LBP-TOP Feature Extraction

Each froth video FV is divided into a froth image sequence $FIS=[I_{1},I_{2},I_{3}\ldots ,I_{n}]$ with *n* images based on time intervals. To extract texture features from these dynamic image sequences, the LBP-TOP algorithm is utilized. The fundamental concept behind this approach is to expand the temporal dimension and segment the image sequence into three planes: XY, XT, and YT, based on their spatio-temporal relationship. Typically, the froth images are perceived as a collection of individual frames in the XY plane. However, in addition to the X and Y directions, the sequence also possesses a temporal dimension along the T-axis. Consequently, an image sequence can be interpreted as a set of YT planes along the X-axis and a set of XT planes along the Y-axis. The XT plane represents the image obtained by scanning each row along the time axis, whereas the YT plane depicts the image obtained by scanning each column along the time axis. It is important to note that the XY, XT, and YT directions are mutually orthogonal.

For a froth image sequence, the XY plane encompasses the visual representation of the froth image itself, whereas the XT and YT planes capture the temporal and spatial dynamics. To extract comprehensive features, the LBP feature sequences are subjected to TOP operations along these three directions. Subsequently, three-dimensional feature mapping sequences, namely, $S_{xy}$, $S_{xt}$, and $S_{yt}$, are obtained as

(3) $$\left\{\begin{array}{c} S_{xy}={\mathrm{TOP}}_{xy}\left(\left\{{\mathrm{LBP}}_{P,R}\left(I_{1}\right),\ldots ,{\mathrm{LBP}}_{P,R}\left(I_{N}\right)\right\}\right)\\ S_{xt}={\mathrm{TOP}}_{xt}\left(\left\{{\mathrm{LBP}}_{P,R}\left(I_{1}\right),\ldots ,{\mathrm{LBP}}_{P,R}\left(I_{N}\right)\right\}\right).\\ S_{yt}={\mathrm{TOP}}_{yt}\left(\left\{{\mathrm{LBP}}_{P,R}\left(I_{1}\right),\ldots ,{\mathrm{LBP}}_{P,R}\left(I_{N}\right)\right\}\right) \end{array}\right.$$

**Figure 3:**
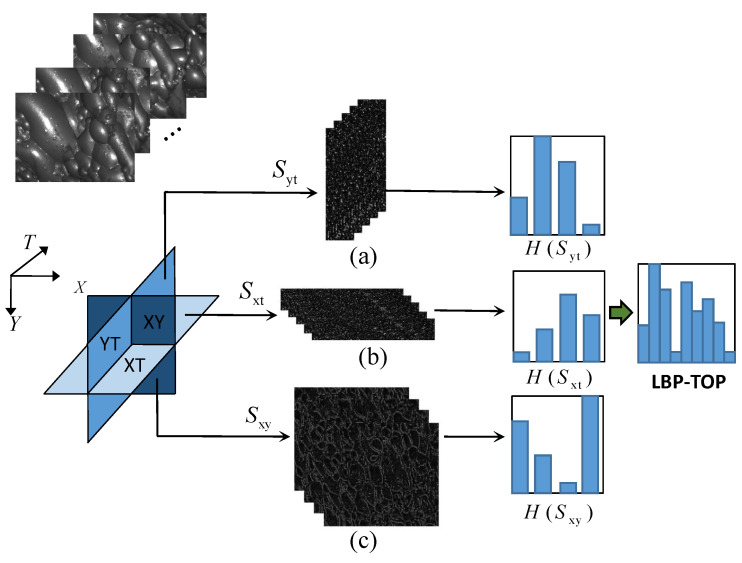
LBP-TOP histogram feature operation procedure.

The TOP operation process of LBP feature mapping sequences in the above three directions is shown in Figure 3, where (a) represents the stack of XY plane on time T axis, (b) represents the stack of XT plane on Y axis, and (c) represents the stack of YT plane on X axis. It can be seen that the XT plane and YT plane contain more time information.

After obtaining the LBP eigenvalues for each direction, the histogram is defined as

(4) $$H_{i}\left(j\right)=\sum_{x,y,t}s\left(f_{i}\left(x,y,t\right)==j\right)$$

where $f_{i}\left(x,y,t\right)$ represents the pixel value of the LBP feature on the *i*th plane, i={XY,XT,YT}, j=0,1,…,255, and the *s* function is defined as

$$s\left(A\right)=\left\{\begin{array}{cc} 1, & \mathrm{if}\;A\;\mathrm{is}\;\mathrm{true}\\ 0, & \mathrm{if}\;A\;\mathrm{is}\;\mathrm{false} \end{array}\right..$$

The cascade LBP histogram features on the three planes, $H_{\mathrm{XY}}$, $H_{\mathrm{XT}}$, $H_{\mathrm{YT}}$; the LBP-TOP histogram feature is obtained as

(5) $$H_{\text{LBP-TOP}}=\left[H_{\mathrm{XY}},H_{\mathrm{XT}},H_{\mathrm{YT}}\right]$$

where the XY plane contains texture information and the XT plane and YT plane contain temporal dynamic information; thus, the LBP-TOP histogram features contain dynamic texture information in spatial domain and time domain.

### 2.3. Supplementary Feature Extraction

To enhance the inclusiveness of image texture information, additional texture features are incorporated into the LBP feature sequence. Following the execution of LBP-TOP in each direction, supplementary features (SFs) such as kurtosis, skewness, and coefficient of variation are extracted. Kurtosis serves as an effective metric for quantifying the flatness of data distribution, while skewness accurately measures the symmetry of image distribution. Moreover, the coefficient of variation objectively reflects the extent of dispersion among data pairs. These features take into account the gray-scale distribution of image sets along each direction, thereby serving as valuable complements to the LBP-TOP feature sequences.

For each bubble image *I* in the image sequence, calculate the coefficient of variation cov(I), kurtosis kurt(I), and skew skew(I) to generate a vector SFs(I)=[cov(I),kurt(I), skew(I)]. Feature extraction is carried out on the image sequence containing *n* images, and an additional feature vector in one direction is obtained, as shown in Equation (6).

(6) $$\mathrm{SFs}\left(S\right)=\mathrm{mean}\left(SFs\left(S_{i}\right)\right),i=1,\ldots ,n.$$

Then, combine additional feature vectors in the three-dimensional direction to obtain $H_{\mathrm{SFs}}$ as shown below.

(7) $$H_{\mathrm{SFs}}=\left[\mathrm{SFs}\left(S_{xy}\right),\;\mathrm{SFs}\left(S_{xt}\right),\;\mathrm{SFs}\left(S_{yt}\right)\right].$$

Finally, the linear splicing method is used to supplement the LBP feature sequence, and the ${\text{LBP-TOP}}_{\mathrm{SFs}}$ feature is obtained.

(8) $$H_{{\text{LBP-TOP}}_{\mathrm{SFs}}}=\left[H_{\mathrm{LBP-TOP}},H_{\mathrm{SFs}}\right].$$

## 3. Multi-View Sequential Feature Extraction

As described in Section 2, the froth image sequences investigated in this paper are selected by framing the froth videos according to the time nodes, which can be regarded as a set of temporal datasets. Extracting features from such sequential image data remains challenging. Inspired by the idea that 2D perspectives from various angles can better identify and represent 3D objects [28], this section introduces a multi-view temporal feature aggregation network model that incorporates a view pooling layer, with a multi-channel ResNet serving as its backbone. Multiple images from the froth image sequence are utilized as inputs to the model, enabling the pre-training and extraction of flotation froth’s multi-view ResNet temporal aggregation features.

### 3.1. Representation Recognition Based on Multiple Views

**Figure 4:**
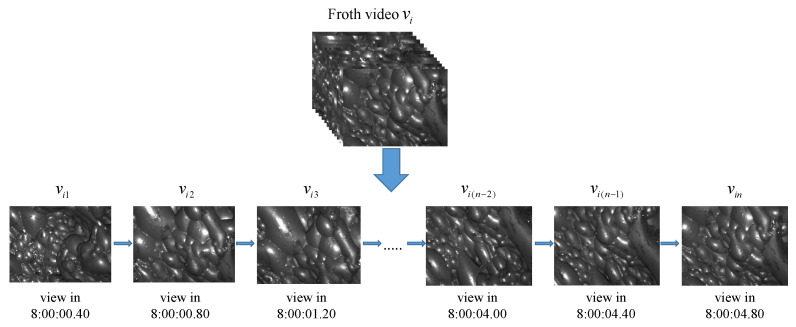
Multiple view description of froth video.

In the field of computer vision, identifying 3D objects conventionally involves building 3D models directly. However, due to the relatively limited number of 3D objects in contact and the complex process of building 3D models, researchers have proposed an alternative approach of inferring 3D objects from 2D images from different perspectives [29]. Multiple views taken at various angles contain rich three-dimensional shape information, leading to a more comprehensive object representation and accurate recognition of the 3D object. Considering the applicability of this idea to the flotation condition identification, the flotation froth image data set used in this paper consists of image sequences selected from sub-frames of froth videos. Figure 4 illustrates a set of temporal froth images $\left[v_{i1},v_{i2},\ldots ,v_{in}\right]$ extracted from a video sample $FV_{i}$ at different time nodes, which can be treated as multiple views of a corresponding froth video. Since flotation conditions are artificially defined and classified based on flotation videos with different characteristics, as described in Section 5, utilizing these flotation time-series images allows for a more accurate identification of the corresponding conditions. This, in turn, enables a more comprehensive description of the complex dynamics of the flotation process.

The multiple views representation of froth temporal image sequences is highly effective for direct utilization in classification tasks. However, it comes with the drawback of generating a large amount of data, leading to a significant reduction in model training efficiency. To address this challenge, a solution is considered to aggregate the feature information of multiple images within each froth image sequence into a more descriptive feature [28]. In this approach, each descriptive feature corresponds to a set of froth image sequences, providing valuable information for classification tasks. This not only integrates the temporal dynamic information but also reduces the dimension to a certain extent, resulting in a considerable improvement in calculation efficiency.

### 3.2. Flotation Multi-View Sequential Feature Model

As previously discussed, the primary focus lies in aggregating multiple views to characterize the temporal features of froth image sequence. In this section, a multi-view temporal feature aggregation network is proposed, which incorporates multi-view CNN operations based on image CNN. Considering that ordinary CNN networks can only extract shallow features of images, deep neural networks are employed to extract deep features. However, as the network deepens, the issue of gradient vanishing or exploding can arise, which can be addressed through normalized initialization and intermediate initialization. Despite these solutions, the model accuracy’s rate may rapidly decline after reaching saturation during convergence, resulting in the degradation phenomenon. To overcome this problem, a variant of CNN, ResNet deep network, is adopted as the convolutional neural network.

**Figure 5:**
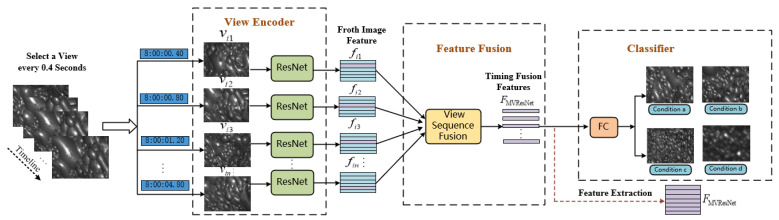
MVResNet temporal feature aggregation network.

The multi-view temporal feature aggregation network, depicted in Figure 5, is based on ResNet. In this network, each image in the froth image view sequence $Fvs=[v_{i1},v_{i2},\ldots ,v_{in}]$ is considered as a separate sample and serves as the input to the network. Feature sequences $Fs=[f_{i1},f_{i2},\ldots ,f_{in}]$ are extracted, respectively, through ResNet convolution operations for each image, and these features are then aggregated using global maximum pooling operations in the pooling layer, as shown in Equation (9). Finally, the aggregated features are sent through the remaining network to achieve recognition and classification tasks.

(9) $$F_{\mathrm{MvResNet}}=\mathrm{MAX}\left(f_{i1},f_{i2},\ldots ,f_{i(n-1)},f_{in}\right).$$

The multi-view temporal feature aggregation network utilizes ResNet with shared parameters among all branches in the initial part of the network. Subsequently, the integration of multi-view features is achieved by employing the View-Sequence-Fusion layer, which takes the maximum value of each element to combine the features of multiple views into one descriptive feature $F_{\max}$. The advantage of View-Sequence-Fusion here is similar to that of Max-Pooling, where dimension reduction is achieved by taking the maximum value of elements. However, the difference lies in the dimensions used for max operation. Common Max-Pooling is a two-dimensional operation carried out on the pixel matrix of an image, while View-Sequence-Fusion performs a three-dimensional maximization operation. Since there are multiple view data, the maximum value of a certain piece of the multiple views is taken as the feature. In this way, each group of froth image sequences corresponds to a more descriptive feature, resolving the issue of inefficient model training caused by an excessive number of feature samples. In summary, the multi-view temporal feature aggregation network consists of the following three main components:

- View Encoder: The View Encoder is responsible for extracting a feature representation for each individual view. In this network, each view has its own encoder based on the ResNet architecture, comprising a convolutional layer, a pooling layer, and a fully connected layer. The View Encoder processes the input data from each view to obtain a meaningful feature representation that captures the distinctive characteristics of that view;
- Feature Fusion Layer: Once the feature extraction is complete for each view encoder, the Feature Fusion Layer is responsible for combining the features from different views. This fusion method involves using either maximum operations or average operations. The maximum operation selects the maximum value for each feature element among the different views, while the average operation computes the average value for each feature element from all the views. These fusion techniques help combine the extracted information from multiple views into a unified and comprehensive feature representation;
- Classifier: The final component of the multi-view temporal feature aggregation network is the Classifier. It takes the fused feature representation generated by the Feature Fusion Layer as input and is responsible for identifying and classifying the target. The classifier uses this integrated feature representation to make predictions and assigns the appropriate labels to the input data based on the learned patterns and relationships within the features. Its role is to determine the flotation condition or working state based on the combined information from multiple views of the froth image sequences.

**Figure 6:**
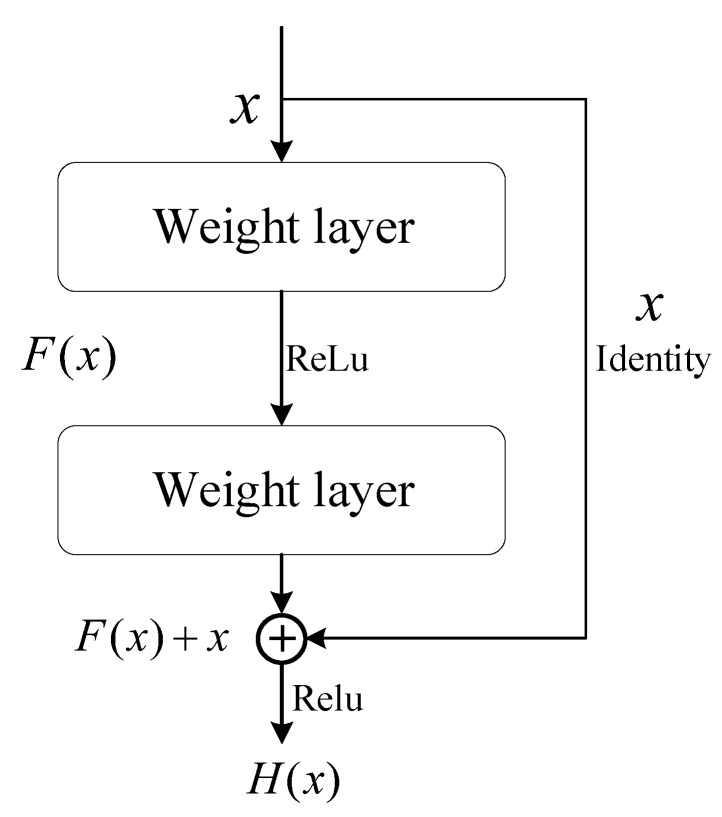
Residual element.

The ResNet convolutional network offers several advantages, including addressing the problem of gradient vanishing, improving network convergence, and enabling deeper networks. Compared to ordinary networks, ResNet introduces a shortcut connection between each pair of layers. As illustrated in Figure 6 [30], this structure no longer forces some stacked layers to directly fit the function F(x) but instead takes the feature map *x* from the upper layer as the initial output of a partial result.

The final output is given by Equation (10); when F(x)=0, it effectively becomes an identity mapping. This design enables the network to learn the residual part H(x)−x, enhancing its robustness. By employing ResNet in the multi-view temporal feature aggregation network, the model’s ability to effectively aggregate features from different views and handle complex dynamics is improved.

(10) H(x)=F(x)+x.

Given that the froth images are captured by a depth camera at a fixed angle, resulting in multiple views selected based on time node frames, the global average pooling operation is considered for aggregation in the view sequence fusion layer. Global average pooling calculates the average value of all the elements in each feature map, effectively summarizing the information from different views into a single representation. By employing global average pooling in the view sequence fusion layer, the model can still effectively integrate information from multiple views and achieve feature aggregation without increasing the computational complexity.

The multi-view temporal feature aggregation network follows a two-step training process. Initially, pre-training is utilized to learn the initial network weights, followed by fine-tuning using the froth image training dataset. This comprehensive training approach enhances the model’s performance and improves its ability to accurately classify froth images, as discussed in Section 5. Compared to the ResNet features extracted from independent single froth images, the aggregation features obtained through the multi-view pooling operation exhibit enhanced descriptiveness and lower dimensions. This reduction in dimensionality enhances the efficiency of classification training, making the model more effective and practical for froth image classification tasks. Extensive verification and testing have demonstrated the efficacy of the proposed approach for accurate flotation condition recognition, as described in Section 5.

## 4. Multi-Modal Sequential Hypergraph Convolutional Networks

The Hypergraph Neural Network (HGNN) represents an advanced feature optimization framework, leveraging hypergraph structures to elucidate the interrelations among data points, thereby achieving enhanced efficiency via hyperedge convolution operations [21]. This model is particularly adept at handling multimodal data, enabling the fusion of disparate data sources through the integration of adjacency matrices to construct hypergraphs. When applied to the domain of flotation condition identification, the HGNN is shown to substantially augment production efficiency.

### 4.1. Graph and Hypergraph

Graph convolutional neural networks (GCN) have attracted much attention in recent years due to their capability to leverage data graph structure, offering advantages over traditional convolutional neural networks in representation learning. GCNs excel at encoding the graph structure of diverse input data and effectively handling irregularly correlated data, making them particularly useful in semi-supervised learning tasks. However, while GCNs establish connections between data structures through pin-to-pair associations, real-world data might not always exhibit simple pair-wise relationships, especially in multi-modal scenarios where data modeling can be more intricate. Consequently, when confronted with higher-order and complex data, convectional graph structures may have limitations in expressing data correlation [31].

**Figure 7:**
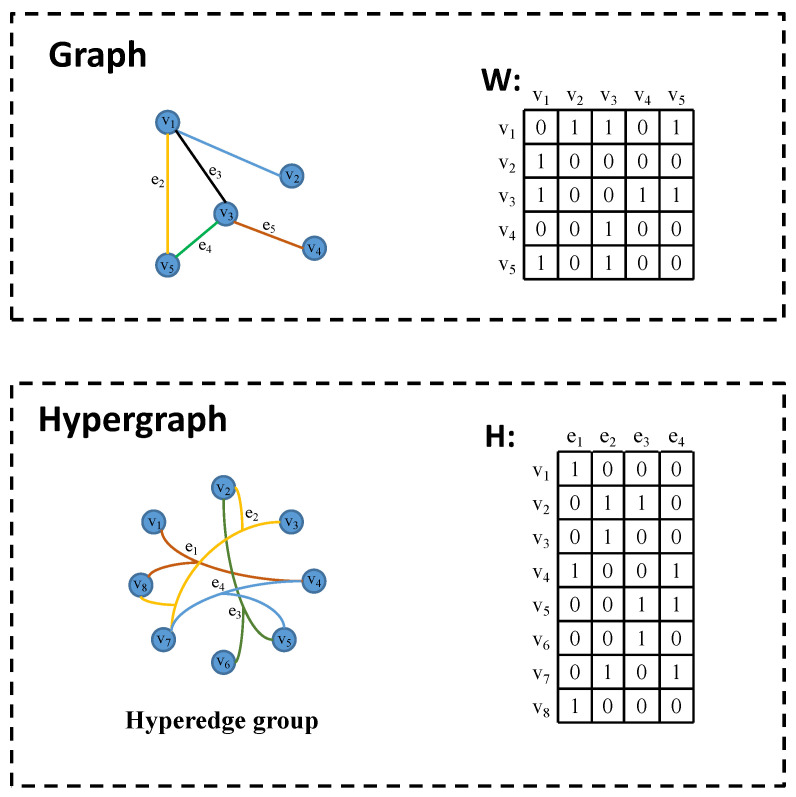
The comparison between graph and hypergraph.

Constructing a hypergraph structure can effectively model higher-order correlations between data. As illustrated in Figure 7, traditional graph structures allow each edge to connect only two vertices, representing a pair-wise association between data elements. In contrast, the hypergraph structure employs infinite hyperedges to encode higher-order data correlations. Each hyperedge can connect two or more vertices, thereby encompassing richer data information. It is worth noting that a simple graph can be considered as a special case of a hypergraph. Furthermore, when dealing with multi-modal or heterogeneous data, multi-modal data can be fused by connecting hypergraph adjacency matrix *H* to facilitate the integration of diverse data modalities.

### 4.2. Hypergraph Convolution

The predominant characteristic of hypergraph neural networks lies in their capacity to encode high-order data correlations. The foundational principle revolves around spectral convolution on hypergraphs, wherein the HGNN layer adeptly extracts higher-order correlations via node-edge-node transformations [21]. This mechanism exhibits a robust capability in data modeling, underscoring the effectiveness of HGNN in capturing complex relational data structures.

The hypergraph structure includes a vertex set *V*, a hyperedge set *E*, and the weight diagonal matrix *W* composed of the weights corresponding to each hyperedge. The definition of hypergraph is as follows:

(11) G=(V,E,W)

where *G* represents a hypergraph instance composed of multiple hyperedges, which can be represented by the association matrix *H* with the element defined as

(12) $$h\left(v,e\right)=\left\{\begin{array}{cc} 1, & \mathrm{if}\;v\in e\\ 0, & \mathrm{if}\;v\notin e \end{array}\right.$$

where vertex v∈V, and its vertex degree is defined as $d\left(v\right)=\sum_{e\in E}w(e)h(v,e)$. The hyperedge e∈E, whose hyperedge degree is defined as $\delta \left(e\right)=\sum_{v\in V}h(v,e)$. In addition, define $D_{e}$ and $D_{v}$ diagonal matrices representing hyperedge and vertex degrees, respectively.

A hypergraph instance *G* is created based on the training data, where the vertices within the hypergraph represent each data sample. The main objective of the classification task is to effectively categorize these vertices effectively. To achieve this, a regularization framework [32] is introduced, as presented in Equation (13), aiming to ensure that the vertex labels exhibit smoothness on the hypergraph structure.

(13) $$\arg\;\min_{f}\left\{R_{emp}\left(f\right)+\Omega \left(f\right)\right\}$$

where $R_{emp}\left(f\right)$ represents the supervised empirical error, f(·) denotes the classification function, and Ω(f) is a normalized loss function and a regularizer on hypergraphs, which is defined as

(14) $$\Omega \left(f\right)=\frac{1}{2}\sum_{e\in E}\;\sum_{\{u,v\}\in V}\frac{w(e)h(u,e)h(v,e)}{\delta (e)}\times {\left(\frac{f(u)}{\sqrt{d(u)}}-\frac{f(v)}{\sqrt{d(v)}}\right)}^{2}$$

Let $\Theta =D_{v}^{-1/2}HWD_{e}^{-1}H^{⊤}D_{v}^{-1/2}$, and Δ=I−Θ, where *I* is the identity matrix. The matrix Δ is semi-definite and is known as the hypergraph Laplacian matrix. The normalized standard loss function is defined as

(15) $$\Omega \left(f\right)=f^{⊤}\Delta .$$

Given a hypergraph with *n* vertices, the hypergraph Laplacian matrix Δ is a semi-definite matrix of size n×n, which can be decomposed into $\Delta =\Phi \Lambda \Phi^{⊤}$, where $\Phi =\mathrm{diag}(\phi_{1},\ldots ,\phi_{n})$ and $\Lambda =\mathrm{diag}(\lambda_{1},\ldots ,\lambda_{n})$. Following the analogy with the frequency domain convolution process of ordinary graphs [33], the convolution in the HGNN is defined by transforming the spatial signal into the frequency domain using Fourier transform, defining the convolution in the frequency domain, and then converting the frequency domain signal back to the spatial domain using Fourier inversion. HGNN convolution in the frequency domain is defined as follows:

(16) $$g*x=\Phi \left(\left(\Phi^{⊤}g\right)⊙\left(\Phi^{⊤}x\right)\right)=\Phi g\left(\Lambda \right)\Phi^{⊤}x.$$

The Laplacian matrix is further decomposed, and the complexity of convolution operation is reduced while ensuring the high-dimensional data correlation of nodes in the hypergraph. Finally, the convolution operation of the HGNN is simplified as follows, where the variable θ is the training parameter of the convolution kernel.

(17) $$g*x=\theta D_{v}^{-\frac{1}{2}}HWD_{e}^{-1}H^{⊤}D_{v}^{-\frac{1}{2}}x.$$

### 4.3. MTHGNN Flotation Condition Recognition Algorithm

The detailed flow of multi-modal temporal Hypergraph neural network (MTHGNN) is as follows:

(1) Step 1: Data Preparation

**Figure 8:**
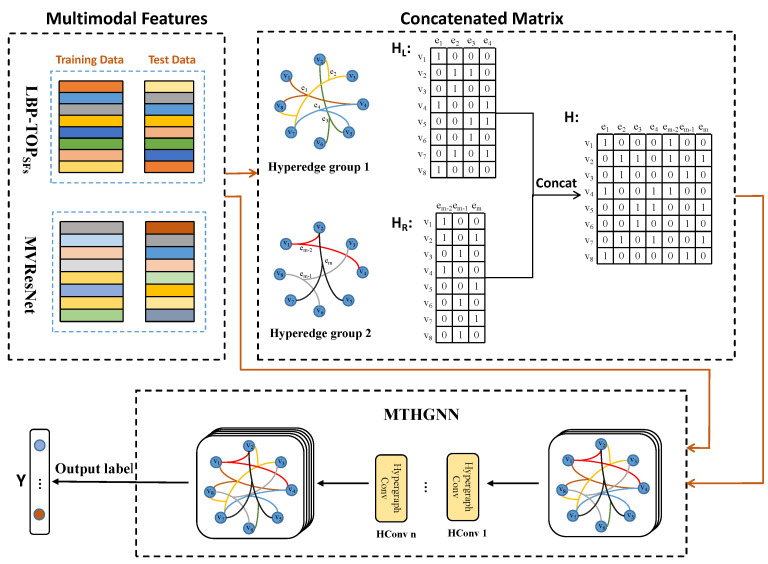
Training based on MTHGNN multi-modal time series data.

Multi-modal bubble feature data were collected, including two modes of ${\text{LBP-TOP}}_{\mathrm{SFs}}$ and ResNet feature data described above. Two kinds of feature extraction were respectively carried out for the image sequence of flotation froth to ensure that the number of samples of the two modes remained consistent. The data are divided into a training set for training the model and a test set for evaluating performance. Multi-modal timing data training based on the MTHGNN is shown in Figure 8.

(2) Step 2: Hyperedge Structure Construction

Each set of froth image sequences corresponds to a feature sample, and each feature sample is used as the vertex of the hyperedge structure. The number of hyperedge connected vertices $K_{L}$ and $K_{R}$ is determined by using the proximity algorithm (KNN). Multiple hyperedge structure groups are constructed based on complex correlations of ${\text{LBP-TOP}}_{\mathrm{SFs}}$ and ResNet multi-modal data sets, respectively. Hyperedge structures capture higher-order relationships and correlations between different data modes.

(3) Step 3: Hypergraph Association Matrices Construction

For each hyperedge structure group, create two hypergraph association matrices, denoted as $H_{L}$ and $H_{R}$. The hypergraph association matrix $H_{L}$ represents the relationship between samples in the internal hyperedge of the ${\text{LBP-TOP}}_{\mathrm{SFs}}$ feature data. The hypergraph association matrix $H_{R}$ represents the relationship between samples in the internal hyperedges of ResNet feature data.

(4) Step 4: Multi-Modal Hypergraph Fusion

Multiple hypergraph correlation matrices $H_{L}$ and $H_{R}$ are concatenated or combined into a total hypergraph *H*, which is realized using Equation (18). The resulting total hypergraph *H* is used as input to train the MTHGNN model.

(18) $$H=[H_{L}\left(K_{L}\right),H_{R}\left(K_{R}\right)].$$

(5) Step 5: Convolution Operation and Training

The HGNN model uses a convolution operation to process the input hypergraph signal $X\in R^{m\times (C_{1}+C_{2})}$. Here, *m* is the number of data samples (hypergraph vertices), $C_{1}$ is the number of attributes of the froth image timing feature ${\text{LBP-TOP}}_{\mathrm{SFs}}$, and $C_{2}$ is the number of attributes of the ResNet timing feature. The convolution operation is defined by Equation (19), where $D_{v}^{-\frac{1}{2}}$ and $D_{e}^{-1}$ are diagonal matrices representing the vertex and edge degrees, respectively. The parameter *W* is the super edge weight matrix, and *H* is the general hypergraph incidence matrix. The training parameter $\Theta \in R^{\left(C_{1}+C_{2}\right)\times C_{3}}$ represents the convolution kernel applied to all vertices on the hypergraph. The result of the convolution operation $Y\in R^{m\times C_{3}}$ is the output label of the multi-modal timing feature of the froth image after training in the HGNN classification task.

(19) $$Y=D_{v}^{-\frac{1}{2}}HWD_{e}^{-1}H^{⊤}D_{v}^{-\frac{1}{2}}X\Theta$$

(6) Step 6: Classification of Flotation Conditions

**Figure 9:**
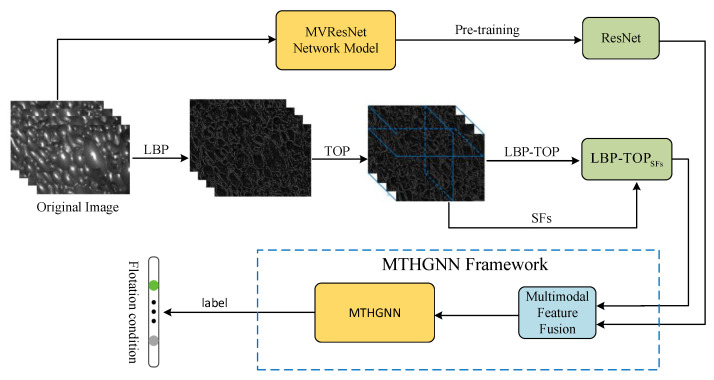
Flotation condition recognition process based on MTHGNN.

The output *Y* represents the classification result of the multi-modal froth feature data. The model learned the fusion information from the characteristic data of ${\text{LBP-TOP}}_{\mathrm{SFs}}$ and ResNet through the hypergraph neural network and could accurately classify the flotation conditions according to the multi-modal timing characteristics of the fusion. The flow chart of flotation condition recognition based on the MTHGNN is shown in Figure 9.

The MTHGNN approach enables effective multi-modal data fusion and classification, utilizing hypergraph neural networks to capture complex correlations between different data modalities and improving the precision of flotation condition classification in froth image analysis.

## 5. Experiment and Discussion

### 5.1. Data Set Description

**Figure 10:**
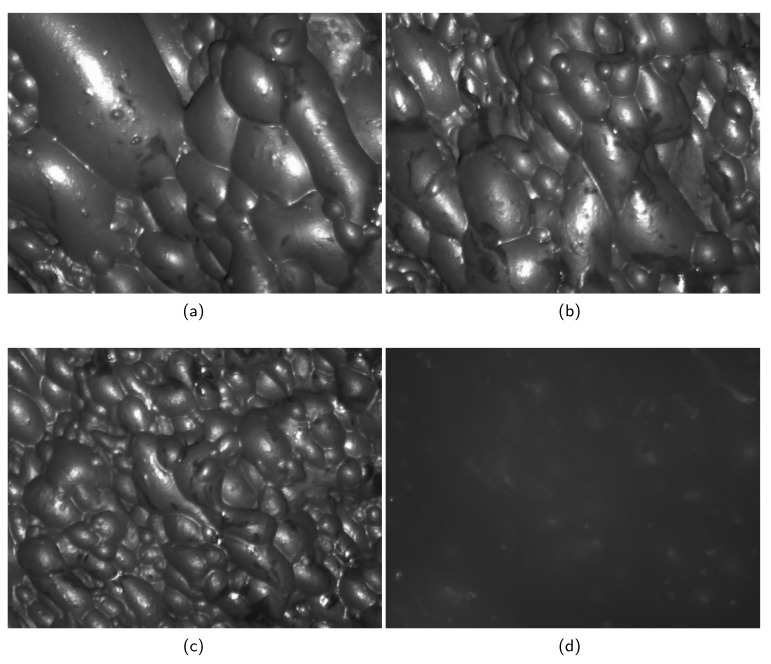
Typical images in the four classes. (**a**) Working condition a. (**b**) Working condition b. (**c**) Working condition c. (**d**) Working condition d.

The open-access dataset [34] utilized in this study originates from a flotation plant situated in southern China. Within the plant, a depth camera has been installed on the flotation machine to continuously monitor the froth state in the flotation cell. Video samples, each lasting for 10 s, were captured by the camera at five-minute intervals. A total of 2373 video samples were collected during the experiment. To create a representative froth image sequence for further analysis, the initial 5 s of each froth video were extracted. Continuous frame sampling was conducted at intervals of 0.4 s within this timeframe. The resulting sampled images were utilized as the froth image sequences and served as the input for the condition recognition model. Subsequently, 2373 froth image sequences were classified by experienced flotation workers, as depicted in Figure 10. The classification process aimed to categorize the sequences into four primary working conditions.

(1) **Working Condition a (660 samples)**: Under this condition, the flotation froth exhibits the following characteristics: large froth size, non-uniform distribution, rough texture, and relatively transparent froth color. The froth samples collected under this condition indicate a relatively low mineral content. Consequently, the flotation efficiency is not significantly high.
(2) **Working Condition b (696 samples)**: Under this condition, the flotation froth demonstrates the following characteristics: a moderate froth size, uniform distribution, relatively clear texture, and a relatively full shape. Additionally, the froth exhibits a relatively high carrying capacity. Under this condition, the froth texture is remarkably good, indicating a high mineral content. Consequently, the flotation efficiency reaches an optimal level.
(3) **Working Condition c (625 samples)**: Under this condition, a majority of the froths appear broken and contain a higher concentration of mud and impurities. This is typically attributed to an excessive amount of chemical agent being present. Such a working condition not only results in the wastage of chemical agents but also negatively impacts the concentrate grade.
(4) **Working condition d (392 samples)**: This is an abnormal working condition, which may cause equipment damage due to some improper operation or the camera cannot focus on the froth surface due to the low liquid level in the tank. In this case, the scraper may not be able to contact a large amount of froth, resulting in low flotation efficiency.

It is evident that the shapes and characteristics of the froth under different working conditions are distinct. In this study, we employed the LBP-TOP algorithm to extract the dynamic texture features and supplementary features from the froth images under each working condition. Furthermore, we constructed a multi-view temporal feature aggregation network, to extract the temporal features representing the aggregation of multiple froth images. These froth feature data were trained as the input of the HGNN to achieve the classification of the froth images. Compared to the manual subjective division of flotation conditions, this intelligent method is more accurate and efficient.

### 5.2. Experimental Parameter Selection

As stated in Section 2, the extraction of texture features from froth images using LBP operators is influenced by the local neighborhood radius *R* and the number of isometric pixels *P*, with *P* having a significant impact on image feature extraction. If the *P* value is too small, the image contrast becomes too low, and if the *P* value is too large, more interference factors affect texture extraction result. Through grid search, we determined the optimal values as R=3 and P=8 for the froth image processing using LBP. Following the original LBP processing of the froth image, the TOP operation is performed. In the froth image sequence, we set the frame number of the continuous froth image sequence as N=12, and the time axis radius as $R_{T}=L$, where L≤(N−1)/2. The L+1th frame of the froth image sequence is taken as the center frame, and the LBP characteristic value is calculated on the XY plane. Subsequently, *L* frames of froth images before and after the center frame are taken on the XT and YT planes, respectively, to calculate the LBP feature values. Finally, the LBP feature values extracted from the XY, XT, and YT directions are concatenated into a one-dimension vector, which represents as the LBP-TOP feature of the current froth image sequence. Considering that the number of frames is much smaller than the pixel value, $R_{T}=1$ is taken, followed by histogram operation to obtain the feature dimension of 768 dimensions. The supplementary features extracted from each direction are concatenated with the LBP-TOP feature value, resulting in a total feature dimension of 777 dimensions.

The multi-view temporal feature aggregation network was pre-trained on the froth image data set. To achieve the optimal performance, several key hyperparameters in the network were set. The training set was configured to encompass 75% of the total sample size. The learning rate lr was set to 0.001, and the attenuation base gamma was set to 0.1. The Stochastic Gradient Descent (SGD) optimizer was utilized with a momentum of 0.9. The total sample size was 2373, and the batch_size of 16 was explored during the training. The model was trained on a GPU for accelerated computation.

**Figure 11:**
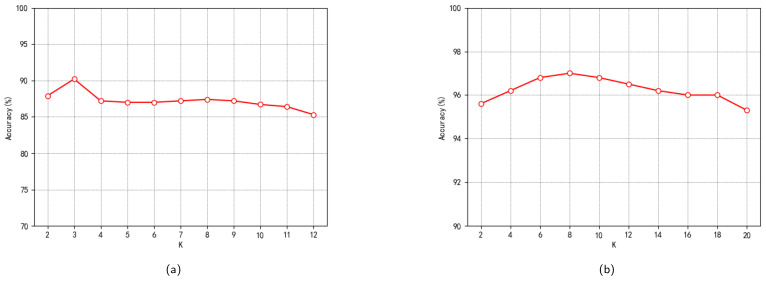
Results of training with different *K* values in two modes. (**a**) On the ${\text{LBP-TOP}}_{\mathrm{SFs}}$ feature. (**b**) On the ResNet50 feature.

The extracted feature data were trained on the HGNN. To achieve higher classification accuracy, several crucial hyperparameters in the HGNN model were specified. The learning rate, denoted as lr, was set to 0.001, and the exponential attenuation base, denoted as gamma, was set to 0.9. For the convolutional layer, the number of hidden units, n_hid, was set to 128. The drop-out rate, denoted as drop_out, was set to 0.5. When constructing hypergraphs, the number of vertices connected by each hyperedge was determined using the K-Nearest Neighbors (KNN) method. The selection of the *K* value is crucial to the model training results. The visualization of training results of the HGNN on ${\text{LBP-TOP}}_{\mathrm{SFs}}$ and ResNet features, along with their fused features under different *K* values, is presented in Figure 11. It can be observed that the corresponding optimals $K_{L}$ and $K_{R}$ are 3 and 8, respectively, when the single-mode feature training accuracy reaches its peak.

### 5.3. Experimental Analysis

The experimental comparative analysis introduced accuracy (Acc) and Macro-$F_{1}$ ($F_{1}$) as evaluation indicators, to assess the performance of the classification results. Accuracy, Acc, commonly used as a quality metric, directly reflects the correctness of the classification outcomes. On the other hand, $F_{1}$ provides a balanced assessment, minimizing the impact of data imbalance in the classification task. Here TP, TN, FP, and FN represent the counts of true positives, true negatives, false positives, and false negatives, respectively, and Classes represents the number of categories. The evaluation metrics are defined as follows: Acc measures the ratio of correctly classified instances (true positives and true negatives) to the total number of instances in the dataset, given by

(20) $$Acc=\frac{\mathrm{TP}+\mathrm{TN}}{\mathrm{TP}+\mathrm{TN}+\mathrm{FP}+\mathrm{FN}}$$

The formula of Macro-$F_{1}$ is as follows:

(21) $$\mathrm{Macro}\;F_{1}=\frac{1}{classes}\sum_{i=1}^{classes}2\times \frac{precision_{i}\times recall_{i}}{precision_{i}+recall_{i}}$$

where for each class, precision is calculated as the ratio of true positives to the sum of true positives and false positives,

(22) $$precision=\frac{\mathrm{TP}}{\mathrm{TP}+\mathrm{FP}}$$

and recall is computed as the ratio of true positives to the sum of true positives and false negatives,

(23) $$recall=\frac{\mathrm{TP}}{\mathrm{TP}+\mathrm{FN}}.$$

$F_{1}$ is indeed the harmonic average of precision and recall, which allows for a more direct reflection of both accuracy rate and recall rate, finding a balance point between the two. As a result, it provides a more comprehensive evaluation of the model’s performance. In general, higher values both Acc and $F_{1}$ indicate better model performance.

The multi-view temporal feature aggregation network uses the froth data set for both pre-training and fine-tuning, enabling the model parameters to reach their optimal state. For training, two deep convolutional networks, ResNet18 and ResNet50, are employed. The results of condition recognition are presented in Table 1. Notably, the classification accuracy surpasses 90%, providing substantial evidence for the network model’s efficacy in accurately identifying flotation conditions.

To validate the effectiveness of the MTHGNN for condition recognition, this paper conducted a comparative analysis using multi-layer perceptron (MLP) [35], Gate Recurrent Unit (GRU) [36], extreme learning machine (ELM) [10], and GCN [33]. To ensure the credibility of the comparisons, the parameters of these methods are fine-tuned to their optimal states. In the case of MLP, the number of hidden units in the two layers is set to 50 and 100, respectively. The ReLu is selected as the activation function. Set the number of hidden units of the GRU to 60 and the number of network layers to 2. For ELM, two key parameters are set as follows: the number of hidden layer neurons is set to 350, and the activation function used is the Sigmoid function Sigmoid. GCN can be regarded as a special case of the HGNN. To conduct image feature training using GCN, two vertices are connected to each edge, allowing for the representation of image features as graphs. The froth image data extracted are then used for multiple training iterations to compute the average accuracy of different methods. The experimental results are summarized in Table 2 for Acc and in Table 3 for $F_{1}$ scores across various characteristics. Owing to the inherent constraints of MLP, GRU, and ELM methodologies in the context of multi-modal data fusion, the data fusion column of the table exhibits null entries.

As indicated in Table 2, the training LBP-TOP with the MTHGNN method proposed in this paper achieves an accuracy of 88.8%, which is notably higher by 12.6%, 23%, and 3% compared to the MLP, GRU, and ELM classification methods, respectively, and slightly better than GCN. By incorporating with the supplementary features (kurtosis, skewness, and coefficient of variation), ${\text{LBP-TOP}}_{\mathrm{SFs}}$ is utilized for training; the classification accuracy using the HGNN further improves the classification training accuracy 90.2%, which represents improvements of 12.8%, 24.0%, 3%, and 0.4% compared to the other four methods.

As presented in Table 3, the $F_{1}$ metric for the MTHGNN model across diverse features remains commendable. Specifically, the $F_{1}$ score achieved with ${\text{LBP-TOP}}_{\mathrm{SFs}}$ features stands at 0.9120, surpassing the score attained utilizing LBP-TOP in isolation. An enhancement in the $F_{1}$ metric is observed with the incorporation of the time series aggregation feature, ResNe50, escalating to 0.9730. Moreover, the apex of $F_{1}$ scores is achieved following the fusion of multi-modal features, registering at 0.9791. Comparatively, the $F_{1}$ metric of the MTHGNN model outperforms alternative methodologies when trained on identical feature sets, thereby affirming the superior efficacy of the MTHGNN model.

**Figure 12:**
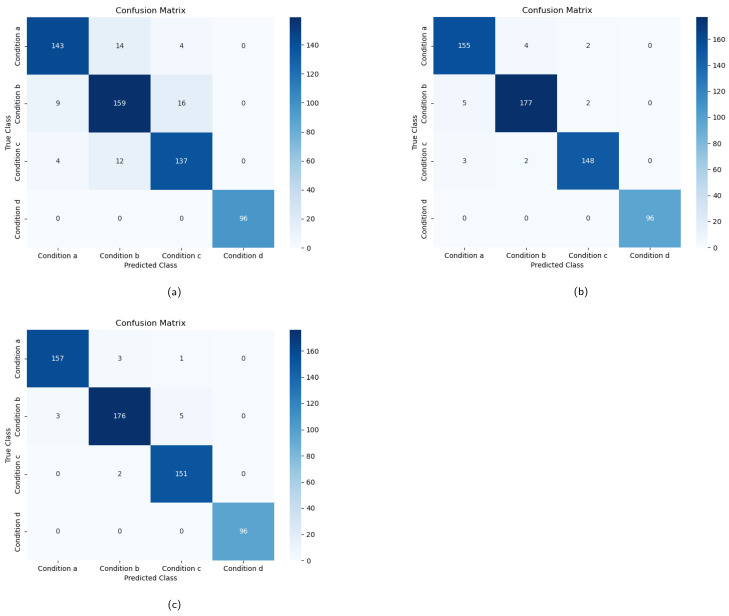
The results of the three modes of condition identification. (**a**) Working condition recognition based on ${\text{LBP-TOP}}_{\mathrm{SFs}}$ feature. (**b**) Working condition recognition based on MVResNet feature. (**c**) Working condition recognition based on LBP-TOPS and MVResNet feature fusion.

To explore multi-modal feature fusion, the MTHGNN merges the ${\text{LBP-TOP}}_{\mathrm{SFs}}$ dynamic texture feature and ResNet50 temporal feature through hypergraph construction, resulting in a remarkable accuracy of 97.7% in classification training. This accuracy surpasses the highest single-model feature training accuracy by 0.7%. Furthermore, the classification accuracy of multi-modal fusion features is also increased by 1.8% compared to GCN, and the corresponding $F_{1}$ score is also the highest. This confirms the idea that the MTHGNN effectively enhances the classification accuracy after fusing multi-modal data. Figure 12 depicts the confusion matrix of the MTHGNN for recognizing three mode conditions, namely, ${\text{LBP-TOP}}_{\mathrm{SFs}}$, ResNet50, and their fusion. The analysis conclusively verifies the effectiveness of the MTHGNN method in training and learning multi-modal characteristics from froth images, enabling accurate condition identification in the flotation process. The experimental results highlight the superiority of the MTHGNN over other traditional classification methods, providing substantial evidence for the method’s efficacy in real-world applications.

## 6. Conclusions

To enhance the efficiency of condition identification in the flotation process, this paper presents a novel approach called the multi-modal temporal hypergraph neural network (MTHGNN). Two novel feature extraction algorithms are introduced: to effectively reflect the dynamic texture feature, the LBP-TOP algorithm extends the froth image sequence from two-dimensional into three-dimensional space, complemented with supplementary features in each plane to enrich the froth description. The multi-view temporal aggregation algorithm performs multi-channel convolution operations on each image in the sequence and integrates the multi-channel features using a multi-view pooling layer. This step enables the extraction of froth’s multi-view temporal aggregation features, effectively capturing the temporal dynamics of froth behavior. To fully correlate the multi-modal feature information obtained, the HGNN is proposed to construct hypergraphs, enabling the encoding of higher-order temporal features. The hypergraph adjacency matrix is used to achieve multi-modal feature fusion, enabling a flexible data modeling approach that significantly improves the classification effect of froth images. Experimental results demonstrate the superiority of the proposed MTHGNN-based method in terms of condition recognition accuracy and stability for froth images in the flotation process compared to existing methods.

Despite the effectiveness of the MTHGNN in enhancing condition identification in flotation processes, it faces limitations such as high computational demands and dependence on extensive labeled data. Future work could aim to reduce these limitations by simplifying hypergraph processing methods and exploring unsupervised learning to lessen the need for labeled data. Additionally, incorporating a broader range of data types could further improve the model’s applicability and performance.

## Figures and Tables

**Table 1:** Training effect of MVResNet.

	Feature	ResNet18	ResNet50
Methods			
MVResNet18		92.7%	-
MVResNet50		-	96.6%

**Table 2:** The Acc of working condition recognition based on various characteristics and different methods.

	Feature	LBP-TOP	${\text{LBP-TOP}}_{\mathrm{SFs}}$	ResNet18	ResNet50	${\text{LBP-TOP}}_{\mathrm{SFs}}$ + MVResNet
Methods						
MLP [35]		76.2%	77.4%	79.2%	84.7%	-
GRU [36]		65.8%	66.2%	80.2%	91.4%	-
ELM		85.8% [10]	87.2%	89.3%	94.2%	-
GCN [33]		88.7%	88.8%	91.7%	95.6%	95.9%
MTHGNN		88.8%	90.2% [24]	92.5%	97.0%	**97.7%**

The best result is marked in bold.

**Table 3:** Different methods on various characteristics of $F_{1}$.

	Feature	LBP-TOP	${\text{LBP-TOP}}_{\mathrm{SFs}}$	ResNet18	ResNet50	${\text{LBP-TOP}}_{\mathrm{SFs}}$ + MVResNet
Methods						
MLP [35]		0.7886	0.7990	0.8139	0.8633	-
GRU [36]		0.6724	0.6849	0.8213	0.9205	-
ELM		0.8744 [10]	0.8893	0.9042	0.9490	-
GCN [33]		0.8979	0.8994	0.9264	0.9670	0.9639
MTHGNN		0.8986	0.9120 [24]	0.9299	0.9730	**0.9791**

The best result is marked in bold.

## Data Availability

The flotation froth image datasets at https://github.com/zunguanF/MTHGNN-Flotationcondition-identification/tree/master (accessed on 26 February 2024).

## Abbreviations

The following abbreviations are used in this manuscript:

HGNN	Hypergraph neural network
MTHGNN	Multi-modal temporal hypergraph neural network
LBP-TOP	Local Binary Patterns based on Three Orthogonal Planes
SFs	Supplementary Feature Extraction
MVResNet	Multi-view ResNet
GCN	Graph convolutional neural networks
